# DNA-Methylation-Caused Downregulation of *miR-30* Contributes to the High Expression of XPO1 and the Aggressive Growth of Tumors in Pancreatic Ductal Adenocarcinoma

**DOI:** 10.3390/cancers11081101

**Published:** 2019-08-02

**Authors:** Asfar S. Azmi, Yiwei Li, Amro Aboukameel, Irfana Muqbil, Philip A. Philip, Ramzi M. Mohammad

**Affiliations:** 1 Department of Oncology, Barbara Ann Karmanos Cancer Institute, Wayne State University School of Medicine, Detroit, MI 48201, USA; 2 Department of Chemistry, University of Detroit Mercy, Detroit, MI 48221, USA

**Keywords:** XPO1, *miR-30*, methylation, pancreatic cancer, proliferation

## Abstract

Pancreatic ductal adenocarcinoma is one of the most aggressive cancers, with high mortality in the United States. One of the important signal transduction proteins involved in the regulation of pancreatic cancer’s aggressive progression is the nuclear export protein (XPO1). High expression of XPO1 has been found in pancreatic, lung, breast and other cancers and lymphomas with a poor prognosis of patients with tumors and high proliferative activity of cancer cells. Because XPO1 exports multiple tumor suppressor proteins simultaneously from the nucleus, the inhibition of XPO1 may retain multiple tumor suppressors in the nucleus, resulting in the suppression of cell proliferation and the induction of apoptosis in tumors. In this study, we found that the high expression of XPO1 in pancreatic cancer cells could be, in part, due to the methylation of the *miR-30* gene, leading to the low expression level of the *miR-30* family. By co-transfection of the XPO1 3′-UTR-Luc target vector with *miR-30* mimic, we found that XPO1 is a direct target of the *miR-30* family. We also observed that the enforced expression of the *miR-30* family inhibited the expression of XPO1, resulting in the suppression of pancreatic cancer growth both in vitro and in vivo. These findings could help to design a novel therapeutic strategy for the treatment of pancreatic cancer by introducing *miR-30* into cancer cells.

## 1. Introduction

Pancreatic ductal adenocarcinoma (PDAC) is one of the most aggressive tumors. It is estimated that 56,770 new cases will be diagnosed and 45,750 patients with pancreatic cancer will die in 2019 [1]. The mortality of pancreatic cancer is increasing. It is expected that pancreatic cancer will become the second leading cause of cancer-related death by 2030 [2]. For all stages combined, the 5-year relative survival of pancreatic cancer is only 9%, which is the lowest survival rate compared to other types of cancer [1]. Therefore, there is an urgent need to design new therapeutic strategies to target important molecules which facilitate pancreatic cancer growth based on the novel molecular mechanism.

The nuclear export protein XPO1 (also known as CRM1) is critical for the control of the nuclear export of proteins, rRNA, snRNA, and some mRNA. More importantly, XPO1 mediates the transport of growth-regulatory proteins including tumor suppressor proteins from the nucleus to the cytosol. It has been found that tumor cells and tissues from various tumors including pancreatic, lung, breast, and other cancers and lymphomas express a much higher level of XPO1 protein or mRNA compared to normal cells and tissues [3,4,5,6,7]. Moreover, the increased expression of XPO1 has been correlated with the poor prognosis of patients with tumors and an increased proliferative activity of cancer cells [8,9,10,11,12]. Because XPO1 exports multiple tumor suppressor proteins simultaneously from the nucleus, the inhibition of XPO1 may retain multiple tumor suppressors in the nucleus, resulting in the suppression of cell proliferation and induction of apoptosis in tumors. Previously, we have found that XPO1 inhibitors can restore the function of multiple tumor suppressive proteins including FOXO3a, p27, Par4 and p73, causing pancreatic cancer cell death in vitro and tumor inhibition in vivo [5]. However, how XPO1 expression is up-regulated in cancer cells is unclear.

The miRNA regulated protein expression is well known to contribute to the aberrant expression of specific proteins in cancers. In recent years, the methylation status of specific miRNA genes has received much attention for their regulation of the aberrant expression of oncogenes and tumor suppressor genes in cancers [13,14,15]. miRNA is a small non-coding RNA which regulates the expression of its target gene. In cells, miRNAs bind the 3’-untranslated region (3′-UTR) of specific target mRNA, leading to either the degradation of mRNA or the inhibition of translation [16,17,18,19]. In this way, miRNAs exert their regulative effects on biological processes and control cell development and growth. In addition, miRNAs also play important roles in the regulation of pathological processes [20]. The aberrant expression of specific miRNAs and their target mRNAs/proteins has been found to promote cancer cell development, growth, migration, invasion and metastasis [21,22]. The abnormal upregulation or downregulation of specific miRNAs in cancer cells could be due to the hypomethylation or hypermethylation of specific miRNA gene DNAs [14,15]. Therefore, it is important to reveal the regulatory mechanism(s) in the DNA methylation/miRNA/mRNA/protein axis for a specific miRNA regulation loop in order to design the novel therapeutic strategy for the treatment of pancreatic cancer. In this study, we report that the upregulation of XPO1 expression in pancreatic cancer is, in part, due to the hypermethylation of the *miR-30* family gene in cancer cells, leading to the downregulation of the *miR-30* family and the upregulation of XPO1. The upregulation of the *miR-30* family can suppress pancreatic cancer cell proliferation and tumor growth in vitro and in vivo through the inhibition of XPO1 expression.

## 2. Results

### 2.1. The *miR-30* Gene Is Hypermethylated in MiaPaCa-2 Pancreatic Cancer Cells 

**Figure 1:**
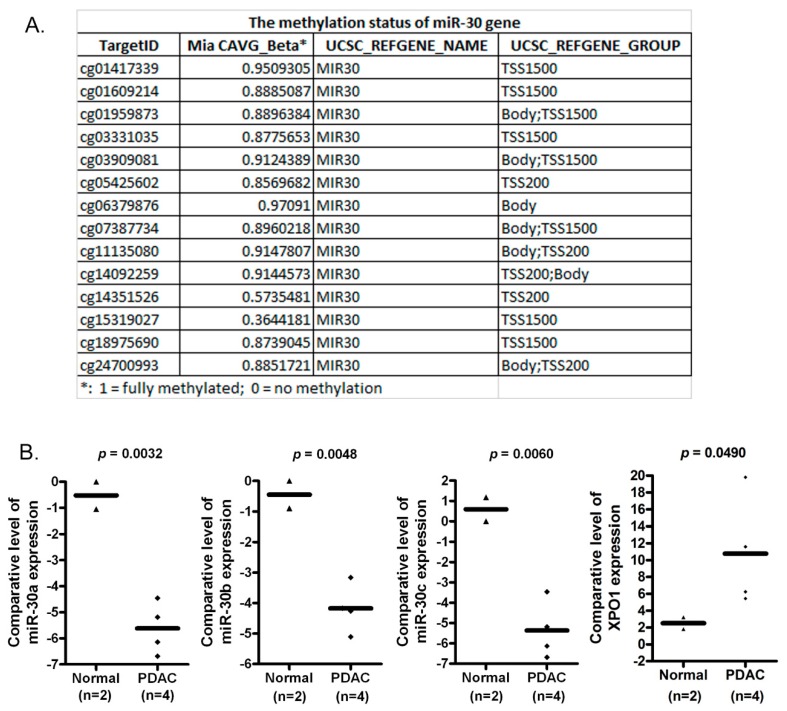
The *miR-30* gene was hypermethylated and *miR-30* expression was down-regulated in pancreatic cancer cells and tissues. Methylation450 chip assay showed the hypermethylation of *miR-30* gene in MiaPaCa-2 pancreatic cancer cells (**A**). The expressions of *miR-30a*, *miR-30b*, *miR-30c* and XPO1 in normal pancreatic epithelial and cancer tissues (**B**) were measured by qRT-PCR.

By using methylation450 chip assay, we found that many DNA sequences (TSS1500, TSS200 and Body) of the *miR-30* gene were hypermethylated in MiaPaCa-2 pancreatic cancer cells (Figure 1A). TSS200 is the region from the transcription start site (TSS) to −200 nt upstream of TSS. TSS1500 covers −200 to −1500 nt upstream of TSS. All these sites are in the promoter, suggesting that the hypermethylation of *miR-30* could reduce the expression level of the *miR-30* family in pancreatic cancer cells.

### 2.2. The Expression of miR-30a, miR-30b and miR-30c Is Lower in PDAC Tissues When Compared with Normal Pancreatic Tissues

Since the hypermethylation of the *miR-30* gene could decrease the expressions of the *miR-30* family, we measured the levels of *miR-30a*, *miR-30b* and *miR-30c* in normal pancreatic epithelial tissues as well as pancreatic cancer tissues. We found that the expression levels of *miR-30a*, *miR-30b* and *miR-30c* were significantly higher in normal pancreatic tissues compared to pancreatic cancer tissues (Figure 1B). These results are consistent with the hypermethylation of the *miR-30* gene (Figure 1A) and suggest that the lower level of the *miR-30* family in pancreatic cancer cells could be due to the hypermethylation in the promoter of the *miR-30* gene in cancer cells.

### 2.3. The Treatment of PDAC Cells with 5-Aza-dC Increases *miR-30* Expression and Decreases the Expression of XPO1

**Figure 2:**
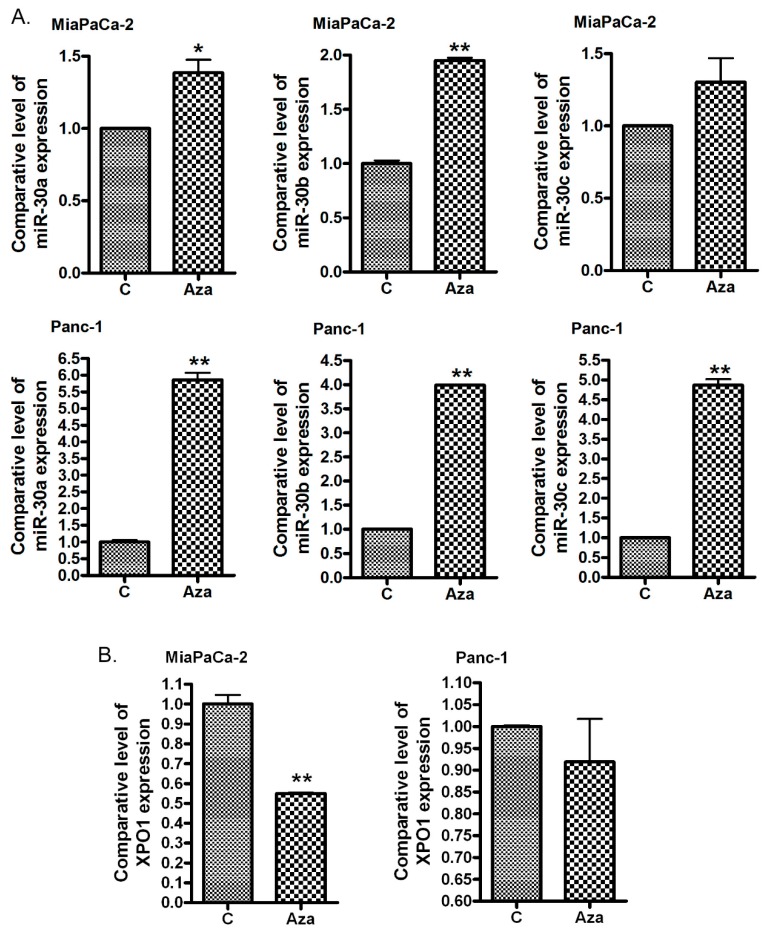
The treatment with 5-Aza-dC increased the level of the *miR-30* family and decreased the level of XPO1 mRNA. MiaPaCa-2 and PANC-1 cells were treated with 5 µM 5-Aza-dC for 5 days. The total RNAs from each sample were extracted and subjected to real-time qRT-PCR for detection of *miR-30a*, *miR-30b*, *miR-30c* (**A**) and XPO1 (**B**) expression (*: *p* < 0.05; **: *p* < 0.01).

In order to confirm that the lower expression of the *miR-30* family was caused by *miR-30* gene methylation, we treated MiaPaCa-2 and Panc-1 cells with 5-Aza-dC, a common DNA methyltransferase inhibitor. We found that 5-Aza-dC treatment increased the expression of *miR-30a*, *miR-30b* and *miR-30c* in both MiaPaCa-2 and Panc-1 pancreatic cancer cells (Figure 2A). Furthermore, we found that the 5-Aaz-dC treatment inhibited the expression level of XPO1 (Figure 2B), which could be a direct target of the *miR-30* family. In addition, we also found that the XPO1 expression was higher in pancreatic cancer tissues compared with normal tissues whereas the *miR-30* level was low in cancer tissues (Figure 1B). These results also suggest that XPO1 could be a target of the *miR-30* family. 

### 2.4. *miR-30a* or *miR-30b* Binds 3′-UTR of XPO1 and Inhibits Its Translational Activity

**Figure 3:**
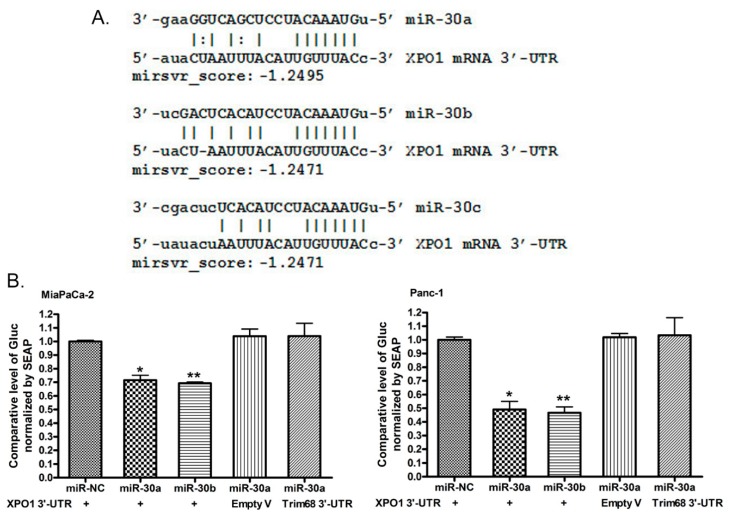
The targeting XPO1 by the *miR-30* family. (**A**) A computerized analysis showed the *miR-30a*, *miR-30b* and *miR-30c* sequence alignment to the sequence of XPO1 3’UTR with a high binding score. (**B**) Co-transfection of the XPO1 3′-UTR luciferase vector, the Empty vector (Empty V) or the Trim68 3′-UTR luciferase vector with *miR-30a*, *miR-30b* mimic, or negative control miRNA showed that *miR-30a* and *miR-30b* directly bonded the 3′-UTR of XPO1 and inhibited its translational activity. (miR-NC: negative control miRNA mimic). (*: *p* < 0.05; **: *p* < 0.01).

In order to investigate whether XPO1 is a direct target of the *miR-30* family, we conducted a computerized analysis of XPO1 3′-UTR sequences and 3′-UTR binding and luciferase analysis. By comparing the computerized prediction of *miR-30* targets with XPO1 3′-UTR sequences, we found that XPO1 could be a target of the *miR-30* family. *miR-30a*, *miR-30b* and *miR-30c* could bind the 3′-UTR of XPO1 mRNA with a high binding score (Figure 3A). To confirm whether *miR-30a* and *miR-30b* could bind the 3′-UTR of XPO1 in cells, we co-transfected the XPO1 3′-UTR target vector, the Empty vector or the Trim68 3′-UTR target vector with *miR-30a*, *miR-30b* or the negative control miRNA mimic into MiaPaCa-2 and Panc-1 cells. Empty and Trim68 vectors served as negative controls. We found that the XPO1 3′-UTR vector luciferase activity was inhibited by the introduction of *miR-30a* or *miR-30b* in MiaPaCa-2 and Panc-1 cells (Figure 3B) while the luciferase activity in the Empty or Trim68 3′-UTR vector was not inhibited. These results suggest that *miR-30a* and *miR-30b* did bind the 3′-UTR of XPO1 mRNA, decrease the level of XPO1 mRNA and inhibit the translational activity of XPO1.

### 2.5. Re-Expression of miR-30a or miR-30b Inhibits the Expression of XPO1

**Figure 4:**
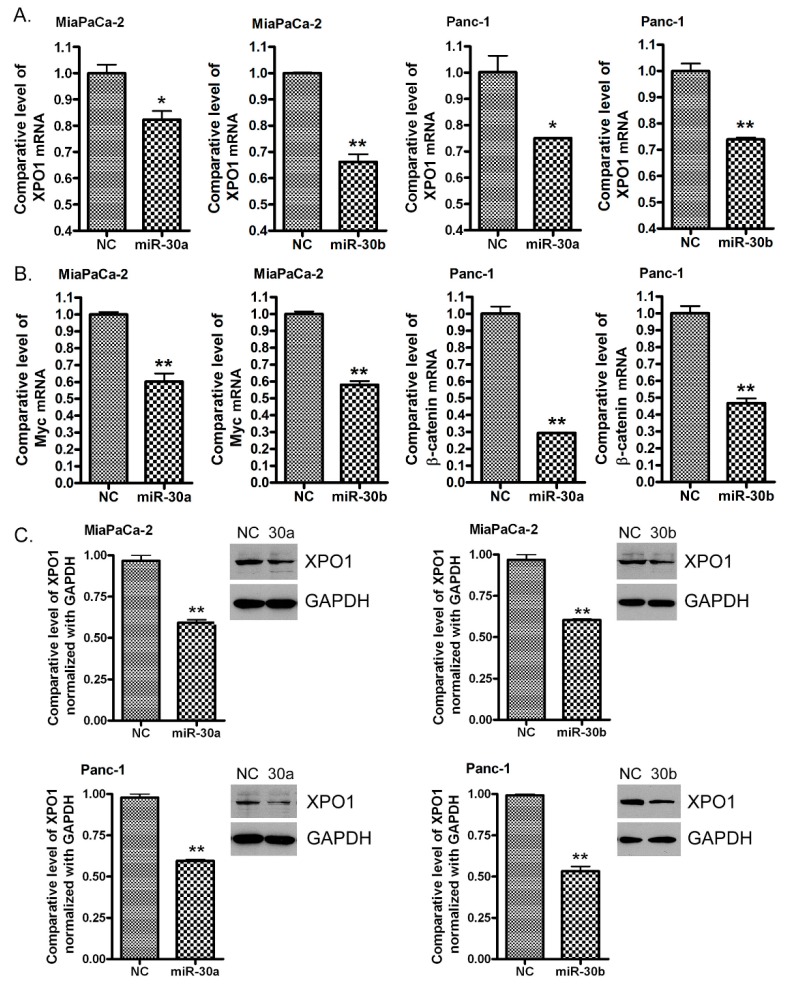
The *miR-30a* or *miR-30b* mimic transfection decreased the levels of XPO1 RNA and protein. MiaPaCa-2 and Panc-1 cells were transfected with *miR-30a* mimic, *miR-30b* mimic, or negative control miRNA for 72 hours. Total RNA was extracted and subjected to real-time qPCR for the detection of XPO1, Myc or β-catenin mRNA (**A**) and (**B**)**.** The total protein was extracted from each sample and subjected to Western Blot analysis for the detection of XPO1 expression at the protein level (**C**). The signal density of the Western Blot analysis was scanned and quantified by AlphaEaseFC. The significance was calculated using the Prism software. (*: *p* < 0.05; **: *p* < 0.01).

To further confirm that XPO1 is a direct target of the *miR-30* family, we transfected the *miR-30a* and *miR-30b* mimics into MiaPaCa-2 and Panc-1 cells. By real-time RT-PCR, we found that *miR-30a* or *miR-30b* transfection decreased the level of XPO1 mRNA (Figure 4A). *miR-30a* or *miR-30b* also decreased the level of Myc and β-catenin mRNAs (Figure 4B), which are known as *miR-30* downstream genes [23,24]. Furthermore, *miR-30a* and *miR-30b* transfection also suppressed the expression of the XPO1 protein (Figure 4C and Figure S2). These results together with the results from XPO1 3′-UTR binding and luciferase analysis suggest that XPO1 is a direct target of both *miR-30a* and *miR-30b*.

**Figure 5:**
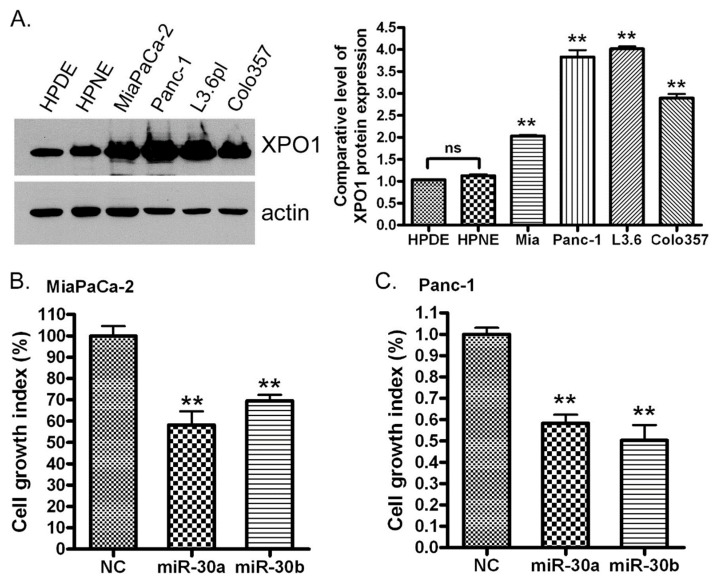
The pancreatic ductal adenocarcinoma (PDAC) cells express a high level of XPO1 and *miR-30* mimic transfection inhibited the growth of pancreatic cancer cells in vitro. (**A**) The total protein was extracted from each cell line and subjected to Western Blot analysis for the XPO1 protein level. The signal density of the Western Blot analysis was scanned and quantified by AlphaEaseFC. The significance was calculated using the Prism software (ns: no significant; **: *p* < 0.01 compared to HPDE). MiaPaCa-2 (**B**) and Panc-1 (**C**) pancreatic cancer cells were transfected with *miR-30a* or *miR-30b* mimic for 9 days and subjected to the MTT assay. The growth index was calculated using the Prism software (*: *p* < 0.05; **: *p* < 0.01).

Since we found the decreased expression of the *miR-30* family in human pancreatic cancer tissues compared with normal pancreatic tissues, we expect that pancreatic cells should overexpress XPO1 if this protein is a direct target of the *miR-30* family. Indeed, we found that the protein expression of XPO1 is significantly higher in MiaPaCa-2, Panc-1, L3.6pl and colo357 pancreatic cancer cells compared with HPDE and HPNE normal pancreatic cells (Figure 5A). In addition, we also found that the expression of XPO1 mRNA in PDAC tissues was higher compared with normal tissues (Figure 1B). Our data were consistent with other investigators’ reports, showing a higher expression of XPO1 in pancreatic and other cancers [3,4,5,6,7]. Similar results from different types of cancers can also be found in the ONCOMINE database (https://www.oncomine.org) (Figure S1).

### 2.6. miR-30a or miR-30b Transfection Inhibits PDAC Cell Proliferation In Vitro

It is well known that the high expression of XPO1 promotes cancer cell growth [8,9,10,25]. In order to evaluate whether the *miR-30* family could control cancer cell growth through the regulation of XPO1 expression, we transfected *miR-30a* and *miR-30b* mimics into MiaPaCa-2 and Panc-1 cells. By measuring the growth index, we found that *miR-30a* and *miR-30b* mimic inhibited the growth of MiaPaCa-2 and Panc-1 pancreatic cancer cells (Figure 5B,C) which have a high expression of XPO1 compared to normal pancreatic HPDE and HPNE cells (Figure 5A and Figure S3). These results together with the results from the mechanistic studies described above suggested that cell growth inhibition by the *miR-30* family could be mediated through the suppression of XPO1 by the *miR-30* family.

### 2.7. The miR-30a or miR-30b Transfection Suppresses PDAC Tumor Growth In Vivo

**Figure 6:**
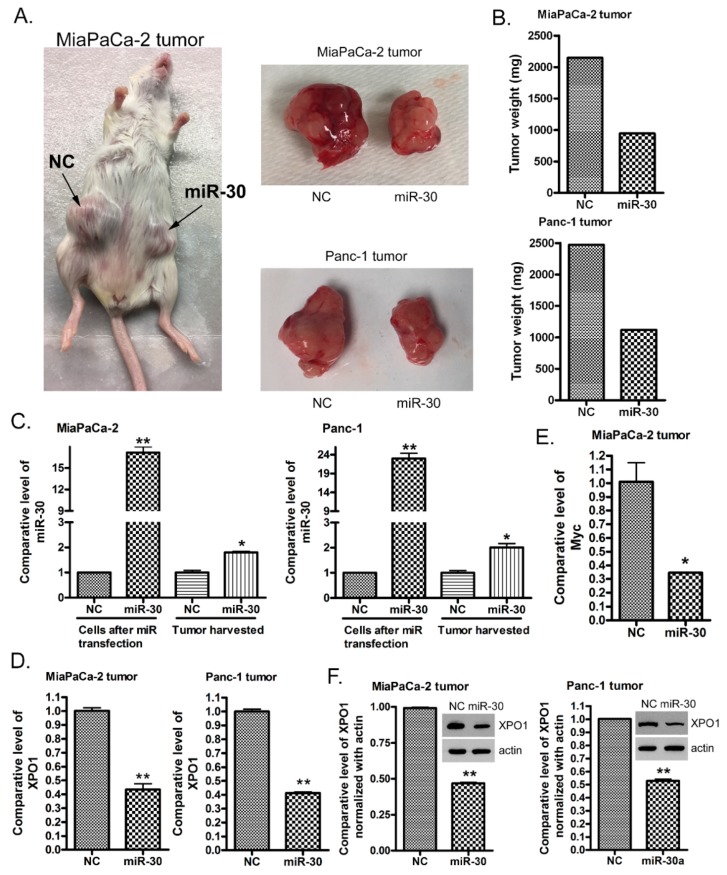
The *miR-30* mimic transfection suppressed tumor formation and growth in vivo through the inhibition of XPO1 expression. MiaPaCa-2 and Panc-1 cells were transfected with *miR-30* mimic or control mimic for 9 days. Then, equal amounts of cells were subcutaneously injected into mice. At the end of the experiment, the tumors were removed and photographed (**A**). Tumor weight was measured at the end of the experiment (**B**). The total RNA from each sample was extracted and subjected to real-time qRT-PCR for the detection of *miR-30*, XPO1 and Myc (**C**–**E**). The total protein from each sample was extracted and subjected to a Western Blot analysis for the level of the XPO1 protein (**F**). The significance was calculated using the Prism software (*: *p* < 0.05; **: *p* < 0.01).

In order to recapitulate the inhibition of cancer cell growth by the *miR-30* family in vivo, *miR-30a* and *miR-30b* mimics were transfected into MiaPaCa-2 and Panc-1 pancreatic cancer cells for nine days. Then, two ICR-SCID mice were subcutaneously injected with the *miR-30* mimic or control mimic transfected MiaPaCa-2 and Panc-1 cells. We found that *miR-30* mimic transfected MiaPaCa-2 and Pan-1 cells formed much smaller tumors and grew slower compared to the negative control mimic transfected MiaPaCa-2 and Panc-1 cells (Figure 6A). The weight of the *miR-30* mimic transfected tumor was only half of the control tumor (Figure 6B). The total RNA was extracted from tumors and subjected to real-time qRT-PCR analysis for *miR-30* and XPO1 expressions. We found that the *miR-30* level was still high while the XPO1 level was significantly downregulated in the tumors formed from *miR-30* mimic transfected MiaPaCa-2 and Panc-1 cells (Figure 6C,D). In addition, the level of another *miR-30* downstream gene Myc was also downregulated (Figure 6E). More importantly, we found that the level of the XPO1 protein was significantly downregulated (Figure 6F and Figure S4). These results suggest that the enforced expression of *miR-30* family suppresses pancreatic cancer growth in vivo through the inhibition of XPO1 expression.

## 3. Discussion

In this paper, for the first time, we reveal an epigenetic mechanism that involves the regulation of nuclear exporter protein XPO1 by the miRNA-30 family. Our results indicate that the hypermethylation mediated silencing of miRNA-30 family results in the upregulation of XPO1. These observations give a unique therapeutic opportunity to target XPO1 through non-coding RNA re-expression.

The epigenetic deregulation of genes has long been recognized as one of the major molecular alterations during the processes of tumor development and progression in humans [26]. Detecting DNA methylation in the promoter of a gene is a common way to understand the epigenetic mechanisms of gene regulation. The DNA methylation status of oncogene and tumor suppressor gene promoters in cancer cells and tissues has been well investigated. It is well known that promoters of many tumor suppressor genes have been hypermethylated while many oncogene promoters have been found to be hypomethylated, leading to the decreased expression of tumor suppressors and the high expression of oncogenes [27]. The aberrant expression of oncogene proteins and tumor suppressors is the cause of tumor development and progression. In addition to the methylation of protein-coding genes, the miRNA gene could also be hypermethylated or hypomethylated in the promoter of the miRNA genes, leading to the altered expression of the miRNA [28]. In line with these observations, we found that the *miR-30* gene was hypermethylated in MiaPaCa-2 pancreatic cancer cells. The hypermethylation occurred mostly in the region of promoters of the *miR-30* gene, suggesting a low expression of the *miR-30* family. Indeed, we observed a decreased expression of the *miR-30* family in pancreatic cancer tissues compared to normal pancreatic tissues. Demethylation agent 5-Aza-dC treatment increased the expression of the *miR-30* family. These results are consistent with the finding that the *miR-30* gene is hypermethylated in pancreatic cancer. The *miR-30* family has been recognized as a tumor-suppressive miRNA in various tumors including multiple myeloma, lung, head and neck, breast, colorectal and other cancers [24,29,30,31,32,33,34,35]. In pancreatic cancers, the *miR-30* family has been found to inhibit EMT, diminish migratory and invasive capacities, and suppress in vivo tumor growth [36]. Therefore, the low expression of the *miR-30* family in pancreatic cancer due to the DNA hypermethylation of the *miR-30* gene in principle could promote tumor development and progression in the pancreas.

It is well known that miRNAs play an important role in the regulation of a large number of target genes that control tumorigenesis and tumor progression [28]. However, how the *miR-30* family functions as a tumor suppressor to inhibit tumor development and progression is unclear. It has been reported that *miR-30* could target Wnt/β-catenin, EGF/Src, Myc, Rab18 and other signaling to inhibit tumor growth [23,24,29,33]. It is well known that one miRNA may have multiple target genes while the expression of target genes is not uniquely regulated by one miRNA. In our study, by the co-transfection of the XPO1 3′-UTR target vector with *miR-30* mimic, we found that XPO1 is a direct target of the *miR-30* family. Therefore, the high expression of XPO1 found in pancreatic cancer and other tumors could be, in part, due to the low expression of the *miR-30* family, which is caused by the hypermethylation of the *miR-30* gene. Moreover, we observed the down-regulation of the XPO1 mRNA expression by the demethylation agent 5-Aza-dC, consistent with our hypothesis that the *miR-30* family is methylated and that XPO1 is a direct target of the *miR-30* family. However, the XPO1 expression could also be regulated by another mechanism such as the promoter regulation by transcript factors, gene mutation, translation regulation and others [37,38]. Therefore, the methylation of the *miR-30* gene could be one of the causes leading to the high expression of XPO1 in pancreatic cancer.

XPO1 has been known to promote tumor growth in various cancers [8,9,10,25]. The high expression of XPO1 in cancer cells is correlated with the aggressive progression and poor prognosis of cancers including pancreatic cancer [8,9,10,11]. We have found that the inhibition of XPO1 significantly suppressed the pancreatic cancer cell growth in vitro and in vivo through retaining tumor suppressors in the nucleus [5,39]. Other investigators have also reported that XPO1 inhibition leads to the suppression of various tumors such as multiple myeloma, lymphoma, lung, breast, ovarian and other cancers [3,40,41,42,43,44]. Because we found that XPO1 is a direct target of the *miR-30* family, we transfected the *miR-30* mimic into pancreatic cancer cells. We did observe the inhibition of MiaPaCa-2 and Panc-1 pancreatic cancer cell proliferation by the enforced expression of *miR-30*. We also observed that the *miR-30* mimic transfected MiaPaCa-2 and Panc-1 cells more slowly form tumors with a lower expression of XPO1 in mice compared with the control mimic transfected cells. These phenomena are consistent with other reports showing that *miR-30* inhibits cancer cell proliferation [24,29,30,34] and that the inhibition of XPO1 suppresses tumor growth [3,40,43]. These observations demonstrate that enforced expression of the *miR-30* family downregulates the expression of its target gene *XPO1*, leading to the suppression of pancreatic cell and tumor growth in vitro and in vivo. These results suggest that introducing the *miR-30* family into cancer cells that have a low level of *miR-30* due to the methylation of the *miR-30* gene could be a novel therapeutic strategy for the treatment of cancers with a high expression of XPO1.

For the reverse of the methylated *miR-30* gene, demethylation drugs could be used to increase the expression level of the *miR-30* family. However, the side-effects and non-specificity of the epigenetic drugs are the major concerns due to the demethylation of a large number of genes. For introducing the *miR-30* family into cancer cells, the synthesized *miR-30* oligonucleotide could be delivered by viral or non-viral vectors. However, the use of viral vectors has been limited because of the safety concerns including immunogenicity and the risk of oncogenic integration and transformation. Non-viral vectors include lipid-based formulations such as liposomes and nanoparticles to facilitate miRNA delivery [44,45]. Growing experimental studies have focused on the introduction of specific miRNAs by systemic oligonucleotide/nanoparticle delivery, which could induce drug sensitivity and inhibit cancer growth, invasion, and metastasis. However, the limitations still exist for the use of this oligonucleotide/vectors system because of several issues such as poor stability, immune system stimulation, off-target effects and delivery efficiency. To overcome these limitations, we are going to conduct experiments using specially designed dendrimer-encapsulated nanoparticles to deliver the *miR-30* family into cells and release *miR-30* within the cells to prevent miRNA degradation in vivo.

## 4. Materials and Methods 

### 4.1. Cell Lines, Tissues, Reagents, and Antibodies 

MiaPaCa-2, PANC-1 and HPNE cells were purchased from American Type Culture Collection (ATCC, Manassas, VA, USA). Human pancreatic duct epithelial (HPDE) cells, human pancreatic cancer L3.6pl and colo357 cells were obtained from the MD Anderson Cancer Center. MiaPaCa-2, PANC-1, L3.6pl and colo357 cells were maintained in DMEM (Invitrogen, Carlsbad, CA, USA) supplemented with 10% fetal bovine serum (FBS), 100 U/mL penicillin and 100 μg/mL streptomycin in a 5% CO2 atmosphere at 37 °C. HPDE and HPNE cells were cultured in DMEM/FBS or a keratinocyte serum-free medium supplied with 5 ng/mL of epidermal growth factor and 50 μg/mL of bovine pituitary extract (Invitrogen). The cell lines have been tested and authenticated in a core facility Applied Genomics Technology Center at Wayne State University. The method used for testing was short tandem repeat (STR) profiling using the PowerPlex^®^ 16 System from Promega (Madison, WI, USA). The normal and pancreatic cancer tissues were collected from surgical samples in the department of pathology at Wayne State University. This human tissue study was approved by the institutional review board/ethics committee of the Wayne State University (2016-029). The compound 5-aza-2’-deoxycytidine (Aza-dC, Sigma, St. Louis, MO, USA) was dissolved in DMSO to make a stock solution of 10 mM. Anti-XPO1 (Santa Cruz, Santa Cruz, CA, USA) and anti-β-actin (Sigma, St. Louis, MO, USA) primary antibodies were used for Western Blot analysis.

### 4.2. Genomic DNA Extraction and Methylation Analysis 

The genomic DNA from MiaPaCa-2 cells was extracted and purified by using the Wizard Genomic DNA Purification Kit (Promega, Madison, WI, USA). The purified DNA was preceded to bisulfate conversion using the EZ DNA Methylation-Gold Kit (Zymo Research, Irvine, CA, USA). The converted DNA was then applied to the HumanMethylation450 BeadChip (Illumina, San Diego, CA, USA). The Illumina Infinium 450K methylation files obtained from the Illumina iScan scanner were uploaded to GenomeStudio (V2011.1, Illumina, San Diego, CA, USA) using the Methylation module. Data were normalized using the Controls Normalization method. Differentially methylated probes were identified using the Illumina Custom Error Model with Benjamini-Hochberg False Discovery Rate correction. A *p*-value for detection of every probe was calculated (Detection *p*-value) and the probes were discarded if this Detection *p*-value was more than 0.05.

### 4.3. RNA Isolation and miRNA Real-Time RT-PCR 

Total RNA from Pancreatic cell lines and tissues were extracted and purified by using the miRNeasy Mini Kit and RNase-free DNase Set (QIAGEN, Valencia, CA, USA) following the protocol provided by the manufacturer. The expression levels of *miR-30a*, *miR-30b* and *miR-30c* in 5-Aza-dC treated or un-treated control PDAC cells were analyzed by using the Universal cDNA Synthesis Kit (Exiqon, Woburn, MA, USA), the specific LNA™ PCR primer set (Exiqon), and SYBR Green RT-PCR Reagents (Applied biosystems, Foster City, CA, USA). The PCR program was initiated by 10 min at 95 °C before 40 thermal cycles, each for 15 s at 95 °C and 1 min at 60 °C. Data were analyzed according to the comparative Ct method and were normalized by RNU44 and RNU1a1 expression in each sample.

### 4.4. mRNA Real-Time RT-PCR 

The expression level of XPO1 in 5-Aza-dC treated or un-treated and miR-control mimic, *miR-30a* mimic or *miR-30b* mimic transfected PDAC cells was analyzed by real-time RT-PCR using High Capacity cDNA Reverse Transcription Kit and SYBR Green Master Mixture from Applied Biosystems. The sequences of primers used were XPO1-F: ACGAGGAAGGAAGGAGCAGT; XPO1-R: CGAGCTGCATGGTCTGCTAA; GAPDH-F: CCACATCGCTCAGACACCAT; GAPDH-R: ACCAGAGTTAAAAGCAGCCCT; 18S-F: GCAATTATTCCCCATGAACG; and 18S-R: GGCCTCACTAAACCATCCAA. The PCR was initiated by 10 min at 95 °C before 40 thermal cycles, each for 15 s at 95 °C and 1 min at 60 °C. Data were analyzed according to the comparative Ct method and were normalized by GAPDH and 18S rRNA expression in each sample.

### 4.5. Western Blot Analysis 

Western Blot analysis was conducted to measure the alterations in the protein expression of XPO1, which could be targets of *miR-30a*, *miR-30b* and *miR-30c*. MiaPaCa-2 and PANC-1 PC cells were treated with 5 µM 5-Aza-dC for 5 days. In a separated experiment, these cells were transfected with miR-control or *miR-30* mimics for 72 hours. After treatment or transfection, the cells were lysed in a RIPA buffer and the protein concentration was measured using a BCA protein assay (PIERCE, Rockford, IL, USA). The proteins were subjected to 10% or 14% SDS-PAGE and electrophoretically transferred to the nitrocellulose membrane. The membranes were incubated with specific primary antibodies and subsequently incubated with secondary antibody conjugated with peroxidase (Bio-rad, Hercules, CA). The signal was detected using the chemiluminescent detection system (PIERCE). The density of the signal in X-ray films was quantified by AlphaEaseFC (Alpha Innotech, San Leandro, CA, USA).

### 4.6. Re-Expression of miR-30 in PDAC Cells 

MiaPaCa-2 and PANC-1 PDAC cells were seeded in 6 well plates and transfected with the miR-control or *miR-30* mimics (Applied biosystems) at a final concentration of 20 nM using the DharmaFact Transfection Reagent (Dharmacon, Lafayette. CO). After 3 days of transfection, the cells were split and transfected repeatedly with the miRNA mimic or control every 3–4 days for the indicated times. The total RNA from each sample was then extracted. One microgram of RNA was subjected to RT-PCR using the High Capacity cDNA Reverse Transcription Kit (Applied Biosystems) and SYBR Green PCR Master Mix (Applied Biosystems), as described earlier. The total proteins from each sample were also extracted and subject to Western Blot analysis as described earlier.

### 4.7. Luciferase Activity Assay for Confirming miRNA Binding to Target 3’UTR 

MiaPaCa-2 and Panc-1 cells were seeded in 96-well plates and incubated for 24 h. The cells were co-transfected with an XPO1 3′-UTR luciferase vector (GeneCopoeia, Rockville, MD), Empty vector (luciferase vector without 3′-UTR from GeneCopoeia) or Trim68 3′-UTR luciferase vector (GeneCopoeia) and *miR-30a*, *miR-30b* or miRNA negative control using DharmaFECT Duo Transfection Reagent (Dharmacon). The vector includes XPO1 3’ UTR target sequence fused downstream to a Gaussia luciferase (GLuc). The vector also contains secreted alkaline phosphatase (SEAP) as an internal control for signal normalization. After 48 h of transfection, GLuc and SEAP activities were assayed using aSecrete-Pair Dual Luminescence Assay Kit (GeneCopoeia) according to the manufacturer’s protocol. The SEAP activity was used as a control when the calculation for the miRNA 3’UTR binding was assessed. 

### 4.8. Cell Growth Inhibition Assay (MTT Assay)

MiaPaCa-2 and HPAC cells were transfected with a miR-control mimic or *miR-30* mimics for 9 days as described. Then, the transfected cells were seeded in 96 well plates. After three days, the cells were subjected to a cell proliferation assay using MTT [3-(4,5-dimethylthiazol-2-yl)-2,5-diphenyltetrazolium bromide]. The spectrophotometric absorbance of the samples was determined by using a plate reader SynergyHT (BioTek, Winooski, WI, USA) at 470 nm. 

### 4.9. Tumor Growth Inhibition Assay 

MiaPaCa-2 and HPAC cells were transfected with a miR-control mimic or *miR-30* mimics (*miR-30a* and *miR-30b*, 1:1) for 9 days as described. Then, the transfected cells were subcutaneously injected into mice. The formed tumors were removed at the end of the experiments and the tumor weight was measured. The total RNA and protein from each tumor were then extracted. One microgram of RNA was subjected to XPO1 and Myc expression analysis by qRT-PCR using the High Capacity cDNA Reverse Transcription Kit (Applied Biosystems) and SYBR Green PCR Master Mix (Applied Biosystems) as described earlier. The RNA from each sample was also subjected to qRT-PCR analysis for *miR-30* as described earlier. The protein from each sample was subjected to Western Blot analysis for XPO1 protein expression. 

### 4.10. Statistics

Wherever appropriate, the data were subjected to a Student’s *t*-test using the GraphPad Prism software (GraphPad Software Inc., San Diego, CA, USA). *p* < 0.05 was considered statistically significant. 

## 5. Conclusions

In conclusion, we demonstrate that there is a regulatory axis (DNA methylation of *miR-30* gene/*miR-30*/XPO1 mRNA and protein) in pancreatic cancer cells. The high expression of XPO1 in cancer cells could be, in part, due to the methylation of the *miR-30* gene, leading to the low expression level of the *miR-30* family which directly regulates the XPO1 expression. Therefore, the enforced expression of the *miR-30* family could inhibit the expression of XPO1, resulting in the suppression of tumor growth in vitro and in vivo. However, additional in vivo studies and clinical trials are needed to evaluate whether the new strategy could be used for the better treatment of pancreatic cancer mediated through the upregulation of the *miR-30* family. XPO1 inhibitors, particularly the Specific Inhibitor of Nuclear Export (SINE) compounds are in several Phase I/II/III clinical studies [46,47,48,49]. The objective response has been observed in patients with metastatic pancreatic cancer that are on a Phase Ib/II clinical study combining Selinexor with gemcitabine and liposome-encapsulated nab-paclitaxel (NCT02178436) [46]. However, since pancreatic cancer is more resistant to chemotherapeutics, cancer could develop a resistance to SINE. In such circumstances, the SINE treatment combined with *miR-30* therapy could decrease the level of XPO1, leading to the synergistic inhibition of tumor growth. In addition, screening patients with the hypermethylated *miR-30* family could allow for the incorporation of these miRNAs to enhance the potency of SINE. These findings could help to design a novel and more effective therapeutic strategy for the treatment of pancreatic cancer.

## Supplementary Materials

The following are available online at https://www.mdpi.com/2072-6694/11/8/1101/s1, Figure S1: High expression of XPO1 in cancers. Figures S2–S4: Western Blot scan, densitometry readings and signal density ratio of XPO1/GAPDH or actin of Figure 4C, Figure 5A and Figure 6F.
