# Supplementary material for: DNA-Methylation-Caused Downregulation of miR-30 Contributes to the High Expression of XPO1 and the Aggressive Growth of Tumors in Pancreatic Ductal Adenocarcinoma
Source: Cancers (Basel). 2019 Aug 2;11(8):1101. doi: 10.3390/cancers11081101 (PMC6721494; doi:10.3390/cancers11081101)
Supplement: Supplementary file 1 [file cancers-11-01101-s001.pdf]

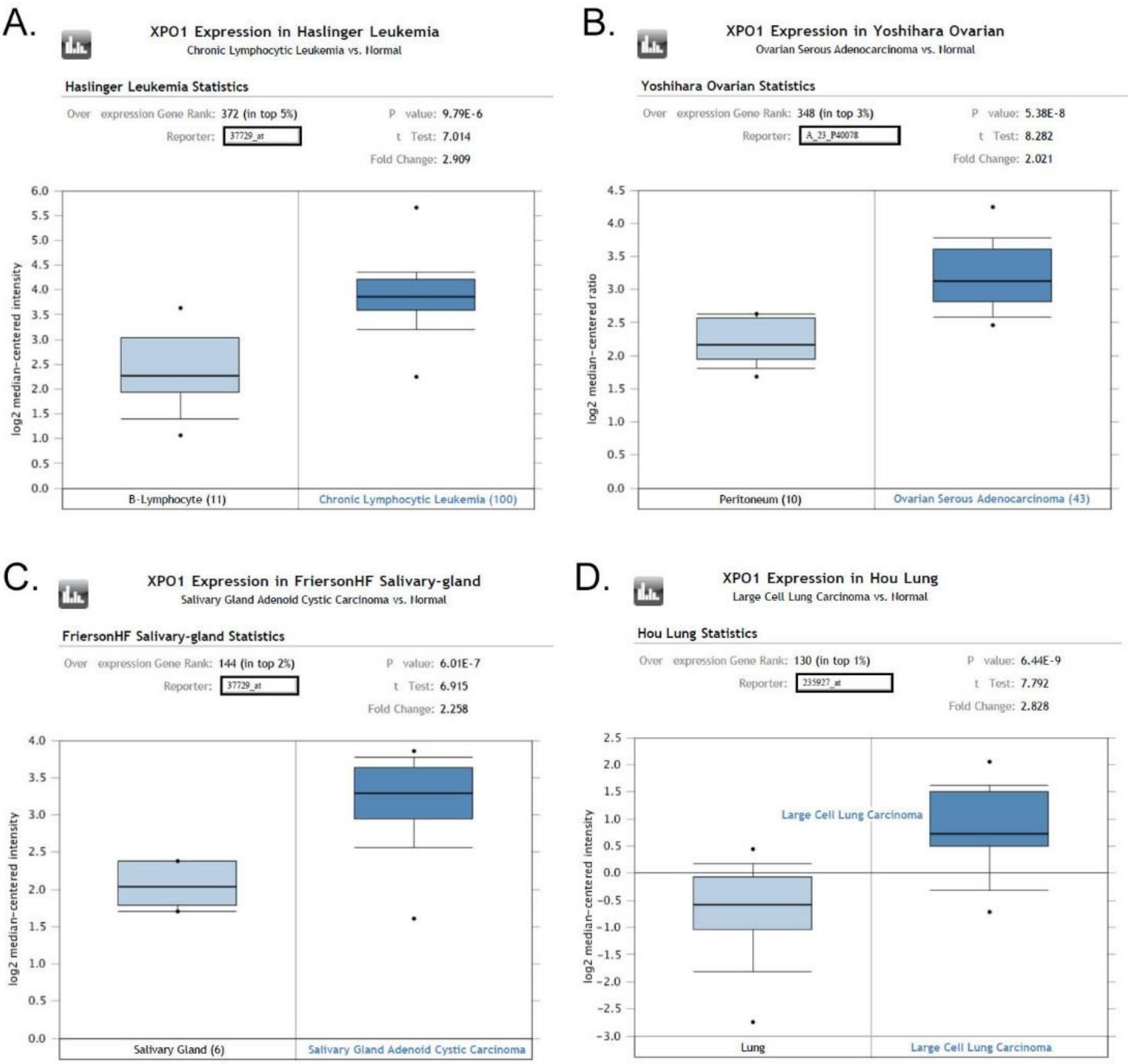

Figure S1. High expression of XPO1 in cancers. Data from Oncomine showed that XPO1 expression was upregulated in various tumors such as leukemia (A), ovarian (B), salivary (C) and lung cancer (D).

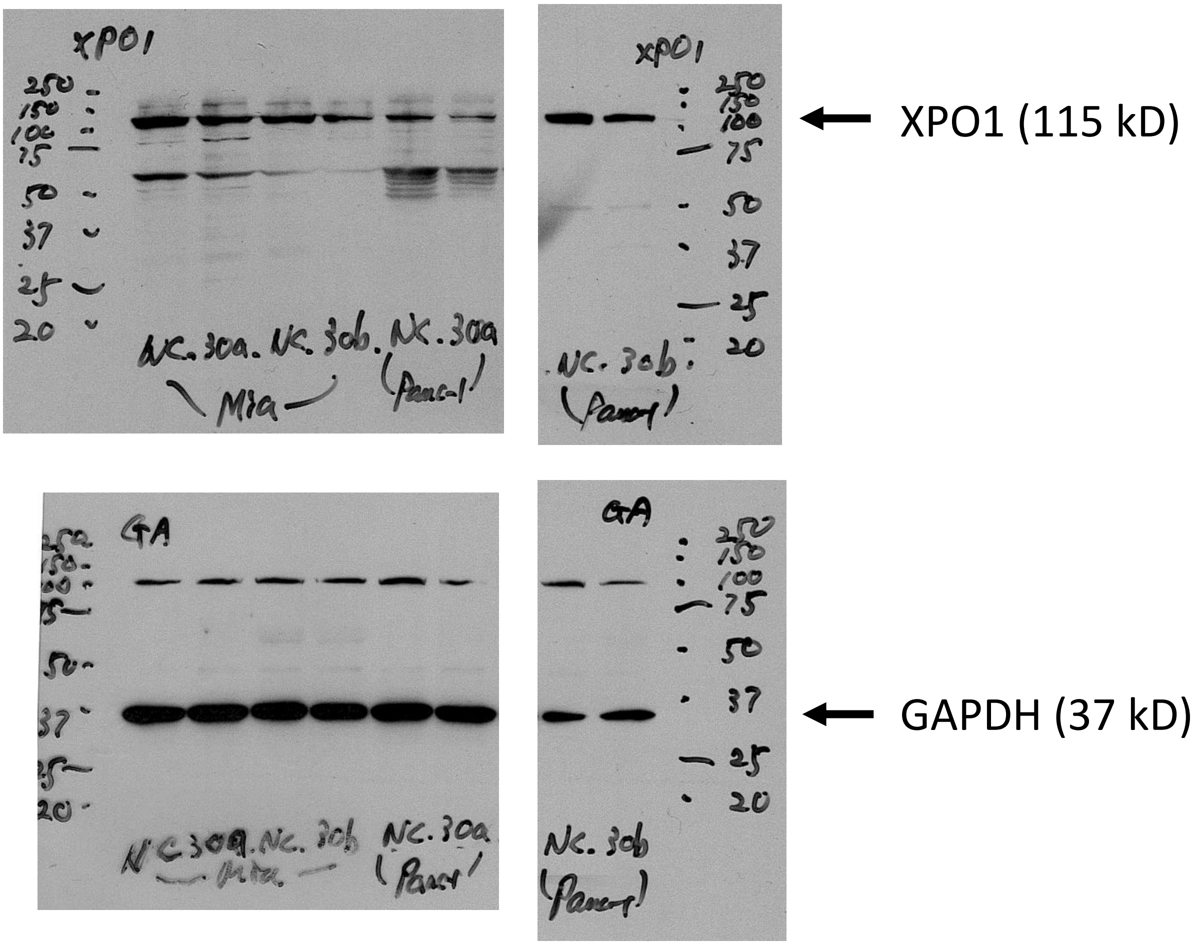

Densitometry readings									
	XPO1					GAPDH			
	NC	miR-30a	NC	miR-30b		NC	miR-30a	NC	miR-30b
MiaPaCa-2 Scan 1	494667	322335	396459	275145		764082	816849	469728	534600
MiaPaca-2 Scan 2	469650	298758	381300	262944		776832	801900	483000	521820
Panc-1 Scan 1	305235	180063	424268	278052		808044	789495	411684	480700
Panc-1 Scan 2	292230	178880	414100	255780		808044	804636	408408	492752

Signal density ratio of XPO1/GAPDH					
	NC	miR-30a		NC	miR-30b
MiaPaCa-2 1	1	0.609527		1	0.609791
MiaPaCa-2 2	0.933844	0.575475		0.935336	0.597023
Panc-1 1	1	0.603776		1	0.561275
Panc-1 2	0.957393	0.588522		0.983863	0.503688

Figure S2. Western Blot scan, densitometry readings and signal density ratio of XPO1/GAPDH of Figure 4C. The Ratios of XPO1/GAPDH were calculated by standardizing the ratios of each control to the unit value.

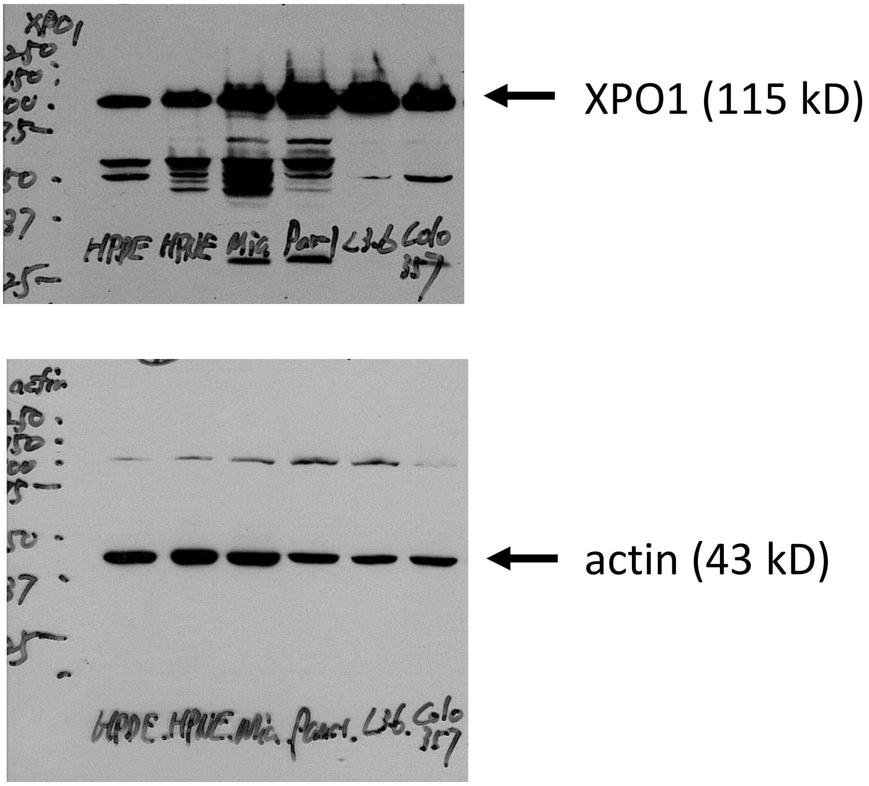

Densitometry readings						
	HPDE	HPNE	MiaPaCa-2	Panc-1	L3.6pl	Colo357
**XPO1**						
Scan 1	499226	553610	1035648	1450944	1264032	924630
Scan 2	495196	574492	1059380	1415200	1253088	937860
**actin**						
Scan 1	488592	504658	504504	364080	310440	329868
Scan 2	475150	495768	527472	385286	316932	314184

Signal density ratio of XPO1/actin						
	HPDE	HPNE	MiaPaCa-2	Panc-1	L3.6pl	Colo357
Ratio 1	1.02176458	1.09700034	2.05280434	3.98523401	4.07174333	2.8030303
Ratio 2	1.04218878	1.15879202	2.00840993	3.67311556	3.95380713	2.98506608

Figure S3. Western Blot scan, densitometry readings and signal density ratio of XPO1/actin of Figure 5A.

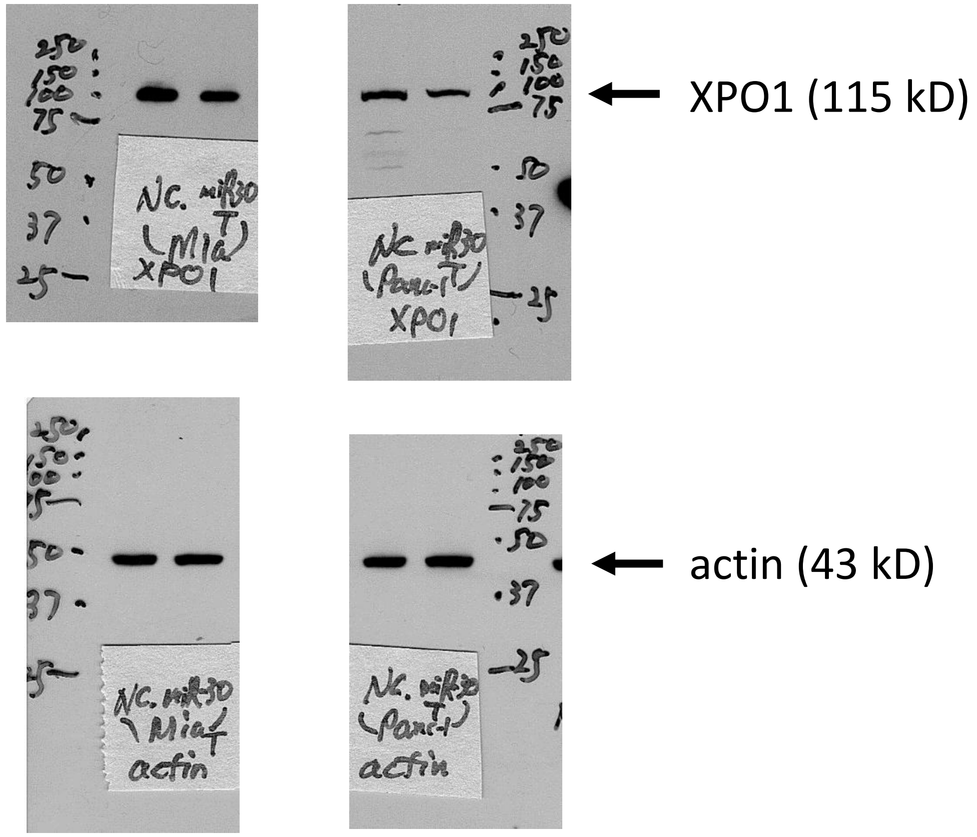

Densitometry readings					
	MiaPaCa-2 Tumor			Panc-1 Tumor	
	NC	miR-30		NC	miR-30
**XPO1**					
Scan 1	896123	425562.4		420028	216972
Scan 2	881121	413819.3		422576	227360
**actin**					
Scan 1	869848	874552		624456	629258
Scan 2	678454	706776		609854	619458

Signal density ratio of XPO1/actin					
	MiaPaCa-2 Tumor			Panc-1 Tumor	
	NC	miR-30		NC	miR-30
Ratio 1	1	0.474893		1	0.516566
Ratio 2	0.983259	0.461788		1.006066	0.541297

Figure S4. Western Blot scan, densitometry readings and signal density ratio of XPO1/actin of Figure 6F. The Ratios of XPO1/actin were calculated by standardizing the ratios of each control to the unit value.
